# Comparing Multivariate with Wealth-Based Inequity in Vaccination Coverage in 56 Countries: Toward a Better Measure of Equity in Vaccination Coverage

**DOI:** 10.3390/vaccines11030536

**Published:** 2023-02-24

**Authors:** Bryan N. Patenaude, Salin Sriudomporn, Deborah Odihi, Joshua Mak, Gatien de Broucker

**Affiliations:** 1 International Vaccine Access Center, Johns Hopkins University Bloomberg School of Public Health, Baltimore, MD 21205, USA; 2 Department of International Health, Johns Hopkins University Bloomberg School of Public Health, Baltimore, MD 21205, USA

**Keywords:** equity, vaccine, immunization, global health, LMICs, health equity, quantitative analysis, socioeconomic, measurement

## Abstract

Introduction: Following a call from the World Health Organization in 2017 for a methodology to monitor immunization coverage equity in line with the 2030 Agenda for Sustainable Development, this study applies the Vaccine Economics Research for Sustainability and Equity (VERSE) vaccination equity toolkit to measure national-level inequity in immunization coverage using a multidimensional ranking procedure and compares this with traditional wealth-quintile based ranking methods for assessing inequity. The analysis covers 56 countries with a most recent Demographic & Health Survey (DHS) between 2010 and 2022. The vaccines examined include Bacillus Calmette–Guerin (BCG), Diphtheria–Tetanus–Pertussis-containing vaccine doses 1 through 3 (DTP1–3), polio vaccine doses 1–3 (Polio1–3), the measles-containing vaccine first dose (MCV1), and an indicator for being fully immunized for age with each of these vaccines. Materials & Methods: The VERSE equity toolkit is applied to 56 DHS surveys to rank individuals by multiple disadvantages in vaccination coverage, incorporating place of residence (urban/rural), geographic region, maternal education, household wealth, sex of the child, and health insurance coverage. This rank is used to estimate a concentration index and absolute equity coverage gap (AEG) between the top and bottom quintiles, ranked by multiple disadvantages. The multivariate concentration index and AEG are then compared with traditional concentration index and AEG measures, which use household wealth as the sole criterion for ranking individuals and determining quintiles. Results: We find significant differences between the two sets of measures in almost all settings. For fully-immunized for age status, the inequities captured using the multivariate metric are between 32% and 324% larger than what would be captured examining inequities using traditional metrics. This results in a missed coverage gap of between 1.1 and 46.4 percentage points between the most and least advantaged. Conclusions: The VERSE equity toolkit demonstrated that wealth-based inequity measures systematically underestimate the gap between the most and least advantaged in fully-immunized for age coverage, correlated with maternal education, geography, and sex by 1.1–46.4 percentage points, globally. Closing the coverage gap between the bottom and top wealth quintiles is unlikely to eliminate persistent socio-demographic inequities in either coverage or access to vaccines. The results suggest that pro-poor interventions and programs utilizing needs-based targeting, which reflects poverty only, should expand their targeting criteria to include other dimensions to reduce systemic inequalities, holistically. Additionally, a multivariate metric should be considered when setting targets and measuring progress toward reducing inequities in healthcare coverage.

## 1. Introduction

Routine vaccination coverage is an essential component of primary healthcare and assessing health systems’ strength. Despite increases in national levels of coverage over time, sub-national inequities in coverage and vaccination status across individuals persist due to multiple structural and socio-demographic barriers to access [1]. Despite this, most metrics used for measuring the degree of inequity in health outcomes, such as vaccine coverage, only allow for measuring disparities along one dimension at a time, such as wealth or urban/rural location [2]. Such measures mask persistent disparities correlated with multiple dimensions. This study utilizes the Vaccine Economics Research for Sustainability and Equity (VERSE) measurement toolkit [3] to compare inequity in full immunization status using both traditional concentration indices and absolute equity gaps (AEG) employing wealth-based ranking with concentration indices and AEGs derived from a multivariate ranking procedure. The analysis is conducted separately for 56 countries utilizing their most recent Demographic and Health Survey (DHS) between 2010 and 2022.

The focus on measuring equity in vaccination coverage derives from a 2017 call by the World Health Organization (WHO) for new methodologies to monitor immunization coverage equity in line with the 2030 Agenda for Sustainable Development. To fill this evidence gap, the Vaccine Economics Research for Sustainability and Equity (VERSE) toolkit was created to provide a standardized approach for measuring and tracking multivariate equity in vaccination coverage, economic impact, and health outcomes [4,5]. The methodology of the VERSE project builds upon existing equity methodologies and toolkits, such as the United Nations Development Programme (UNDP) Global Dashboard for Vaccine Equity, as well as the WHO Health Equity Assessment Toolkit (HEAT) [2,3,6], by expanding the outcomes assessed and by providing a standardized approach for ranking individuals across multiple factors influencing equity including socioeconomic, demographic, educational, sex-based, and geospatial covariates. The metrics produced exhibit several desirable properties of equity metrics such as being comparable over time and between settings, while also being sensitive to the intersectional nature of health equity.

The VERSE toolkit’s approach to assessing equity accounts for the intersectionality of individual and district-level correlates of disadvantage in becoming vaccinated is aligned with approaches taken by numerous governmental institutions and international organizations, including the European Commission [7], the United States Census Bureau [8], the government of the United Kingdom [9], and the United Nations [10], which have all begun expanding beyond a singular focus on income or wealth as the basis for measuring and tracking social equity. However, in examining equity in healthcare access, the measurement of equity remains limited to approaches employing either a single factor for ranking or a series of separate bivariate equity assessments [11,12,13,14]. While this type of sub-group comparison over specific factors is commonplace, a systematic approach for combining and measuring multivariate inequality over multiple groups is needed to produce numbers that better capture the combined magnitude of different types of inequities, while accounting for overlap and intersectionality. For example, urban/rural status and socioeconomic status may partially capture the same type of inequity, but an individual possessing both low socioeconomic status and living in a rural area may also face a higher aggregate degree of disadvantage compared with being of either low socioeconomic status or from a rural area alone [14,15].

In addition to generating comparable equity metrics across 56 countries, this study also compares both multivariate and traditional concentration indices and the corresponding absolute equity gaps for vaccination coverage within the same survey for each country in order to assess whether there are systematic differences in the magnitude of inequity captured between approaches. The analysis is conducted over coverage of 8 key routine vaccines against 4 antigens: Bacillus Calmette–Guerin (BCG), Diphtheria–Tetanus–Pertussis-containing vaccine doses 1 through 3 (DTP1–3), polio vaccine doses 1–3 (Polio1–3), and the measles-containing vaccine first dose (MCV1), as well as an indicator for being fully immunized for age with each of these vaccines.

## 2. Materials & Methods

The data for this study include the most recent DHS survey between 2010 and 2022 for 56 countries (see Appendix A). DHS surveys are nationally representative and all contain data at the individual-level on coverage for eight key routine vaccines against four antigens, which are utilized in this assessment. The vaccines assessed include: Bacillus Calmette–Guerin (BCG), Diphtheria–Tetanus–Pertussis-containing vaccine doses 1 through 3 (DTP1–3), polio vaccine doses 1–3 (Polio1–3), and the measles-containing vaccine first dose (MCV1), as well as an indicator for being fully immunized for age with each of these vaccines. Data on vaccination coverage, as well as socio-demographic covariates, are used alongside the VERSE multivariate vaccination equity assessment toolkit to measure both wealth-based and multivariate equity in vaccination coverage within each country over each vaccine outcome. A complete list of variables from the DHS surveys that are used in the multivariate equity assessment is presented in Appendix B.

The primary outputs of the VERSE toolkit and the featured outcomes of this study are a multivariate concentration index, a relative measure of equity, and an absolute equity gap in coverage, an absolute (level) measure of equity. These measures are derived from literature on the measurement of socioeconomic equity by Wagstaff and Erreygers, combined with measures of “direct unfairness”—a term borrowed from social choice theory, which has been applied to healthcare access in the works of Fleurbaey, Schokkaert, Cookson, and Barbosa [15,16,17,18,19,20,21]. The multivariate concentration index takes the form of a traditional concentration index over vaccination coverage where, instead of ranking individuals by income, individuals are ranked by multivariate unfair disadvantage in access. Multivariate unfair disadvantage, as parameterized in the VERSE model, is measured as an individual-level propensity score for unfair disadvantage, netting out the effect of fair sources of variation in coverage. For the purposes of this study, the only fair source of variation in coverage status is whether a child is underage to receive the vaccine according to the national immunization schedule of the country examined. Unfair sources of variation included in this assessment are the sex of the child, maternal education level, socioeconomic status derived from the DHS wealth index, coverage by health insurance, urban or rural designation, and geopolitical sub-unit of residence. These factors were chosen based on standardized and near-universal data collection across all demographic and health surveys (DHS) [22]. Complete mathematical details of the quantification of unfair disadvantage, as well as the multivariate equity metric produced by the VERSE toolkit, can be found in the VERSE toolkit’s methodological publication [3].

In addition to the multivariate concentration index produced in the VERSE Toolkit, an absolute equity gap is also produced [19,20]. The AEG is a measure of the absolute difference in vaccination coverage achieved by the top 20% compared with the bottom 20% of the population, where the population is ranked based on their propensity score for unfair disadvantage. Mathematically, this is equivalent to isolating the top and bottom quintiles from the Lorenz curve used to estimate the Wagstaff (direct) concentration index [20]. In most equity studies, socioeconomic status as measured by either income or, in the case of the DHS surveys, wealth index, is the sole variable used to rank or group individuals prior to computing a concentration index, slope index, Gini coefficient, Kakwani index, Atkinson index, absolute equity gap, or relative equity gap. In keeping with this convention, we also compute the Wagstaff (direct) concentration index, as well as the AEG between the top and bottom quintile, utilizing the DHS’s wealth index as the only criterion to rank individuals. Concentration indices and AEGs derived from both the multivariate and traditional approaches are computed for 56 countries utilizing the same DHS dataset. The concentration indices and AEGs are then compared directly within countries with one another to provide empirical evidence of the degree of inequity, stemming from multiple factors known to be related to disadvantage in being vaccinated, that is missed by using only the traditional approaches for equity measurement.

## 3. Results

### 3.1. Full Immunization for Age

Among the 56 countries included in the analysis, the average multivariate concentration index for the fully immunized for age status was 0.125 (95% confidence interval: 0.109, 0.140), not weighting by population size. Meanwhile, the average wealth-based concentration index was estimated only at 0.014 (0.004, 0.024)—a difference of 0.110, representing that traditional concentration indices captured, on average, 89% less inequity compared with multivariate concentration index (see Table 1).

The countries with the most significant difference in concentration index between the two approaches were Chad (0.31), Gabon (0.26), Afghanistan (0.25), Angola (0.25), Ethiopia (0.24), Nigeria (0.22), Papua New Guinea (0.21), Yemen (0.20), Guinea (0.19), and Madagascar (0.18). These countries also have among the lowest full immunization coverage of countries with eligible DHS surveys, ranging from 16% to 50%, and the highest multivariate concentration indices, ranging from 0.205 to 0.331 (see Table 2). When considering wealth-based concentration indices, most of these countries either indicate very slight inequity, or none at all. However, comparing the two types of concentration indices illustrates that, among this group of countries, the traditional wealth-based concentration index misses between 67% and 107% of the coverage inequity for full immunization for age.

Furthermore, nine of these ten countries had the largest AEG values in the data set, ranging from a 33 to 59 percentage point gap in coverage between the most and least advantaged quintiles. The differences between the multivariate and wealth-based AEGs range from 3 to 36 percentage points, highlighting the importance of including multiple criteria when assessing disadvantage and equity.

Countries presenting modest differences between concentration indices and equity gaps were typically also among those with the highest levels of coverage for the fully immunized for age status (ranging from 55.6% and 92.1%). While high coverage is likely to be correlated with higher levels of equity utilizing either wealth-based or multivariate approaches—due to fewer individuals missing out on vaccines—it is not always true that a higher performing country will have a higher degree of equity. For instance, Pakistan achieved a full immunization for age coverage level of 65.1% in 2016, yet its multivariate concentration index indicates significant inequity: 0.152, which is 0.123 points higher than its corresponding wealth-based concentration index. Additionally, low coverage does not always lead to inequity, depending on how that coverage is distributed with respect to the assessed characteristics. For example, Uganda achieved a full immunization coverage level of 50.9% in 2016, and yet presented significantly lower multivariate and wealth-based concentration indices, estimated respectively at 0.092 and −0.044, compared with Pakistan. This indicates that while there is a large proportion of children who did not receive the full course of immunization as per Uganda’s immunization schedule, these children are more randomly distributed throughout the population in terms of both geographic and socio-demographic parameters (sex, wealth, education, insurance status) than in Pakistan.

Examining the absolute equity gaps using the multivariate metric, full immunization coverage among the bottom quintile of the population would need to increase by approximately 28.8 percentage points (95% confidence interval: 25.1, 32.6) to achieve a similar level of the fully immunized for age status as the most advantage quintile of the population (see Table 1). When utilizing only the wealth-based approach, the AEG for the fully immunized for age status was estimated as only a 13.8 percentage point gap (95% confidence interval: 9.5–18.2). This indicates that wealth-based measures significantly underestimate the fully immunized coverage gap between the most and least advantaged by 15.0 percentage points, on average, across all datasets (see Table 1).

### 3.2. Individual Vaccines (BCG, DTP, Polio, and MCV)

Focusing on BCG, the eight countries reporting the greatest difference between the multivariate and wealth-based concentration indices are the Maldives (0.178), Afghanistan (0.172), Chad (0.170), Senegal (0.133), Yemen (0.121), Guatemala (0.110), and Madagascar (0.117). However, absolute differences in the AEG vary widely from 1 to 42 percentage points. In contrast, countries with the lowest differences between multivariate and wealth-based concentration indices also had the lowest absolute differences between AEGs. For 44 of the 56 countries in this analysis, the multivariate concentration index is statistically significantly greater than that of wealth-only. For the remaining 12 countries, which include the Kyrgyz Republic, Republic of Congo, Mozambique, Comoros, Benin, India, The Gambia, Sierra Leone, Lesotho, Peru, Malawi, and Ghana, there is no statistical difference between multivariate and wealth-based concentration indices. When looking at total country averages for individual vaccines, BCG has the lowest difference between multivariate and wealth-based estimates with a concentration index difference of 0.046 and an AEG difference of 8.8 percentage points, suggesting that wealth accounts for a significant proportion of the total inequity in this birth-dose vaccine (see Table 1).

If we consider MCV1, the greatest differences in concentration index values are attributed to Guinea (0.230), Afghanistan (0.215), Madagascar (0.166), Angola (0.152), Nigeria (0.145), Ethiopia (0.145), and the Maldives (0.144). Again, we observe a wide range in the differences in AEG values between approaches, ranging from 4 to 39 percentage points. By evaluating inequity with a multivariate approach, it is revealed that the use of a wealth-only ranking metric results in a significant underestimation of inequity for 51 of the 56 countries considered. Countries for which the multivariate concentration index is not statistically different from the wealth-only concentration index include the Kyrgyz Republic, Mozambique, Republic of Congo, Comoros, and Lesotho. Using national averages, the difference between concentration indices as measured by each approach for MCV1 was 0.068 with an AEG difference between approaches of 10.1 percentage points.

For the three-dose vaccines DTP and Polio, the absolute difference between concentration indices generally increases for subsequent doses, though the same trend does not apply to differences in the AEG, suggesting that much of the inequity present after receiving the first dose occurs in the middle of the distribution rather than the tails of the distribution. The greatest difference in DTP concentration index values when comparing the multivariate and wealth-only methodologies are exhibited by Chad (DTP1: 0.192, DTP2: 0.216, and DTP3: 0.268) and Afghanistan (0.186, 0.204, and 0.224). Of all the vaccines included in this study, DTP3 has the highest national average absolute difference between concentration index types at 0.084 and experiences an AEG difference between approaches of 12.9 percentage points, on average. The concentration index differences for DTP1 and DTP2 are 0.053 and 0.066, respectively, with AEG differences between approaches of 9.9 and 11.7, respectively.

The greatest differences between multivariate and wealth-based concentration indices for Polio occur in the Maldives (0.160), Afghanistan (0.130), and Senegal (0.130) for dose 1; Gabon (0.150), Afghanistan (0.142), and Madagascar (0.135) for dose 2; and Angola (0.184), Chad (0.179), and Guinea (0.177) for dose 3. The average differences in concentration index over all countries for Polio doses 1, 2, and 3 are 0.046, 0.059, and 0.080, respectively, with differences in AEG between approaches estimated to be 10.5, 12.4, and 14.5, respectively.

## 4. Discussion

This case-study application of the VERSE toolkit to 56 countries demonstrates that using multivariate procedures for measuring vaccine coverage equity results in significantly larger values compared with traditional methods in most settings. The findings indicate that metrics which only utilize socioeconomic status as a basis for measuring inequity, in order to track whether or not access is pro-poor, will miss a significant amount of the variation in the overall equity in vaccination status that is directly correlated with observable characteristics such as education, sex, and geographic location [23,24].

In countries such as Chad, Afghanistan, or Guinea, if inequities in fully immunized status were only captured through the traditional wealth-based concentration indices or absolute equity gaps, the measures would show that there was no systematic inequity in vaccine coverage within the country (concentration indices between −0.006 and 0.020); however, the multivariate concentration index demonstrates otherwise.

Several recent studies on equity also support the empirical findings of this study. A 2022 systematic review by Ali et al. found that besides wealth, maternal education, sex, and geographic access can also systematically and independently affect vaccination coverage [25]. Additionally, a 2020 study by Acharya et al. comparing the inequalities in full vaccination coverage based on maternal education and wealth quintiles also found that in four of the six studied countries, the absolute inequalities arising from a metric using maternal education level were significantly larger than those measured using wealth quintile [26]. These studies further emphasize the importance of utilizing multivariate metrics to holistically measure and work toward reducing systemic inequality.

Multivariate indicators integrating these multiple socio-demographic parameters effectively quantify differences in coverage even in countries with more modest inequity, such as Uganda. Uganda achieved large increases in overall vaccination coverage during the 2000s with its immunization program through the implementation of Family Health Days and other regular health outreach initiatives, which made the coverage distribution significantly pro-poor. However, when considering the other factors included in the VERSE toolkit’s approach, we can estimate a residual inequity driven by both supply- and demand-side factors such as the district of residence and maternal education [27]. Such an approach revealed aspects of access to vaccines, such as sufficient health literacy and adequate and timely supply across districts, which can help the country consider new approaches to continue to improve coverage equity [28,29].

While the VERSE approach and toolkit can yield a stable metric to track equity over time or between settings, it is also subject to several practical limitations common to all measures of equity and inequality [15]. The first is the inability to objectively state what a “good” or “bad” level of inequity is using the concentration index alone. Like all concentration indices, the results of the VERSE methods lend themselves more toward assessing relative performance than to categorizing objective performance. Although values closer to 0 are objectively preferred, whether a value of 0.1 is bad or good depends upon the circumstances of a specific setting, the mean level of coverage obtained in the setting overall, and the specific benchmarks associated with the rollout and distribution of each vaccine. For this reason, all equity metrics should be put into the context of the outcome or intervention they are evaluating. To assist with this contextualization, the VERSE toolkit produces an absolute equity gap alongside the concentration index to assist with interpretation. While the AEG is a measure of absolute inequity, and the concentration index measures relative inequity, they are both based on the same ranking procedure. They can therefore complement one another, with the AEG providing important coverage-level context to the concentration index.

Another limitation is the data used to populate the tool. While DHS surveys are designed to be nationally representative, evidence shows that settings like urban slums, conflict areas, and refugee settlements are significantly under-sampled, in addition to being more likely to be under-immunized [30]. As a result, estimates of vaccination coverage generated using the DHS are likely to be systematic overestimates of true immunization coverage, and estimates of coverage inequity are likely to be systematic underestimates of true coverage inequities.

## 5. Conclusions

Most measures of equity employed in healthcare equity analyses only examine inequities in outcomes across one dimension which is often decomposed into multiple dimensions. This approach results in the systematic underestimation of aggregate inequity in health outcomes and makes it impossible to measure aggregate inequity across multiple dimensions (e.g., sex, district, and socioeconomic status) in a manner that is comparable across time and place. The VERSE toolkit generates measures of multivariate inequity in vaccination coverage that allow for standardized measurement over time and between locations. Comparing the multivariate concentration indices and absolute equity gaps with traditional wealth-based measures of inequity demonstrates that wealth-based measures systematically underestimate the gap between the most and least advantaged in specific vaccination coverage, as well as fully-immunized coverage. Furthermore, these differences are directly attributable to differences in maternal education, geography, and sex. Not accounting for these multiple dimensions when measuring equity results in a missed vaccination coverage gap between the most and least advantaged of between 1.1–46.4 percentage points, depending on the country. As a result, closing the coverage gap between the bottom and top wealth quintiles is unlikely to eliminate the persistent socio-demographic inequities in both vaccination coverage and access to vaccines linked with other routinely measured covariates. The results suggest that pro-poor interventions, as well as campaigns and programs utilizing needs-based targeting which reflects poverty, should expand their targeting criteria to include other dimensions in order to reduce systemic inequalities, holistically. Additionally, a multivariate metric should be considered when setting targets and measuring progress toward reducing inequities over time and comparing inequity across settings.

## Figures and Tables

**Table 1:** Average inequities among 56 studied countries, by vaccine.

Vaccine	Coverage	Multivariate Concentration Index	Wealth-Based Concentration Index	% Captured Inequity Difference	Coverage Gap Multivariate (Percentage Points)	Coverage Gap Wealth (Percentage Points)	Additional Coverage Gap (Percentage Points)
MCV1	0.772	0.079 (0.067, 0.090)	0.011 (0.002, 0.020)	86.1%	21.5 (17.8, 25.1)	11.4 (7.4, 15.4)	10.1
Polio1	0.860	0.049 (0.039, 0.059)	0.003 (−0.007, 0.013)	93.9%	19.0 (16.3, 21.8)	8.5 (5.5, 11.5)	10.5
Polio2	0.797	0.065 (0.053, 0.077)	0.006 (−0.004, 0.016)	90.7%	22.1 (18.9, 25.2)	9.7 (6.3, 13.2)	12.4
Polio3	0.684	0.087 (0.075, 0.100)	0.007 (−0.003, 0.016)	91.9%	24.0 (20.4, 27.5)	9.5 (5.6, 13.4)	14.5
BCG	0.868	0.058 (0.049, 0.068)	0.012 (0.002, 0.022)	79.3%	23.1 (20.7, 25.4)	14.3 (11.9, 16.6)	8.8
DTP1	0.844	0.063 (0.053, 0.072)	0.010 (−0.001, 0.020)	84.1%	22.8 (20.0, 25.5)	12.9 (9.9, 15.8)	9.9
DTP2	0.789	0.078 (0.066, 0.088)	0.012 (0.003, 0.022)	84.6%	25.0 (22.0, 28.1)	13.3 (9.9, 16.8)	11.7
DTP3	0.716	0.098 (0.086, 0.111)	0.014 (0.005, 0.024)	85.7%	27.1 (23.7, 30.4)	14.2 (10.4, 18.0)	12.9
FULL	0.559	0.125 (0.109, 0.140)	0.014 (0.004, 0.024)	88.8%	28.8 (25.1, 32.6)	13.8 (9.5, 18.2)	15.0

**Table 2:** Inequities in fully immunized status, by country.

Country	Year	Coverage	Multivariate Concentration Index	Wealth-Based Concentration Index	Difference in Equity Levels (Concentration Indices)	Captured Inequity Difference (Percent)	Coverage Gap Multivariate (Percentage Points)	Coverage Gap Wealth (Percentage Points)	Additional Coverage Gap (Percentage Points)
Afghanistan	2015	41.0%	0.24 (0.23, 0.25)	−0.01 (−0.01, 0)	0.25	102.50%	54 (52, 55)	18 (16, 20)	35.7
Angola	2015	30.5%	0.32 (0.31, 0.34)	0.07 (0.07, 0.08)	0.25	77.1%	45 (42, 47)	42 (38, 46)	3.2
Armenia	2015	74.1%	0.06 (0.02, 0.10)	−0.09 (−0.13, −0.05)	0.15	244.0%	21 (14, 29)	−13 (−21.2, 4.8)	34.0
Bangladesh	2016	74.7%	0.04 (0.03, 0.05)	−0.01 (−0.02, 0)	0.05	125.6%	14 (10, 17)	10 (6, 13)	4.0
Benin	2017	60.7%	0.13 (0.11, 0.14)	0.08 (0.08, 0.09)	0.04	34.4%	38 (35, 41)	28 (25, 31)	10.2
Burkina Faso	2010	69.9%	0.08 (0.07, 0.09)	−0.02 (−0.03, −0.01)	0.09	119.0%	27 (23, 30)	16 (12, 20)	11.0
Burundi	2016	76.0%	0.05 (0.04, 0.05)	0 (−0.01, 0.01)	0.05	104.4%	17 (15, 20)	−2 (−5, 2)	19.0
Cambodia	2014	78.4%	0.07 (0.06, 0.09)	0.03 (0.01, 0.05)	0.04	58.1%	30 (27, 34)	17 (13, 20)	13.8
Cameroon	2012	47.7%	0.17 (0.15, 0.19)	0.05 (0.04, 0.06)	0.12	69.6%	37 (33, 41)	33 (29, 38)	4.0
Chad	2014	23.1%	0.33 (0.31, 0.34)	0.02 (0.02, 0.02)	0.31	93.9%	37 (34, 39)	13 (11, 16)	23.2
Comoros	2012	48.4%	0.18 (0.16, 0.20)	0.09 (0.08, 0.10)	0.08	47.0%	39 (34, 44)	25 (20, 31)	14.0
Congo (DRC)	2013	38.2%	0.20 (0.19, 0.22)	0.05 (0.05, −0.05)	0.15	77.0%	35 (32, 38)	26 (22, 29)	9.0
Cote d′Ivoire	2012	44.5%	0.19 (0.17, 0.21)	0.06 (0.05, 0.07)	0.13	70.2%	42 (37, 46)	30 (24, 35)	12.0
Dominican Republic	2013	60.4%	0.07 (0.06, 0.07)	0.01 (0, 0.02)	0.05	81.5%	19 (13, 25)	11 (4, 18)	8.0
Egypt	2013	49.0%	0.04 (0.03, 0.05)	−0.04 (−0.05, −0.04)	0.09	202.4%	10 (8, 13)	8 (6, 11)	2.1
Ethiopia	2016	38.2%	0.28 (0.25, 0.31)	0.04 (0.03, 0.06)	0.24	84.2%	59 (56, 63)	38 (34, 41)	21.9
Gabon	2012	15.9%	0.24 (0.19, 0.29)	−0.02 (−0.03, 0)	0.26	107.5%	21 (17, 24)	−1 (−6, 3)	22.0
Ghana	2014	73.9%	0.06 (0.04, 0.07)	0.02 (0.02, 0.03)	0.03	60.0%	18 (13, 23)	8 (3, 13)	9.8
Guatemala	2014	71.4%	0.04 (0.04, 0.04)	−0.07 (−0.07, −0.06)	0.10	275.7%	12 (10, 15)	6 (4, 9)	5.8
Guinea	2018	33.2%	0.21 (0.19, 0.23)	0.02 (0.01, 0.03)	0.19	91.9%	33 (29, 38)	22 (18, 27)	11.0
Haiti	2016	33.6%	0.22 (0.2, 0.24)	0.04 (0.03, 0.05)	0.18	81.7%	33 (28, 37)	29 (24, 35)	3.7
Honduras	2011	83.1%	0.02 (0.01, 0.03)	−0.05 (−0.06, −0.04)	0.07	323.8%	9 (7, 11)	1 (−2, 3)	8.2
India	2020	48.0%	0.09 (0.08, 0.09)	0.02 (0.02, 0.02)	0.06	75.3%	22 (21, 23)	5 (4, 6)	16.8
Indonesia	2017	60.5%	0.11 (0.1, 0.12)	0.01 (0, 0.02)	0.10	89.6%	35 (32, 38)	14 (11, 17)	21.5
Jordan	2017	60.4%	0.05 (0.04, 0.06)	−0.05 (−0.07, −0.03)	0.09	202.2%	18 (14, 21)	−5 (−11, 1)	22.3
Kenya	2014	67.4%	0.07 (0.07, 0.07)	−0.03 (−0.03, −0.02)	0.10	135.2%	29 (27, 31)	13 (11, 16)	15.2
Kyrgyz Republic	2012	67.1%	0.09 (0.07, 0.11)	0.04 (0.03, 0.05)	0.05	60.0%	27 (22, 31)	−20 (−25, −15)	46.4
Lesotho	2014	70.9%	0.06 (0.06, 0.07)	0.04 (0.03, 0.04)	0.03	40.3%	22 (14, 29)	16 (9, 24)	5.5
Liberia	2019	53.3%	0.1 (0.08, 0.11)	0.03 (0.02, 0.03)	0.07	74.2%	27 (21, 32)	15 (8, 21)	12.2
Madagascar	2021	50.1%	0.21 (0.19, 0.22)	0.03 (0.02, 0.03)	0.18	87.8%	47 (44, 50)	31 (27, 34)	16.3
Malawi	2015	74.5%	0.04 (0.03, 0.04)	0.02 (0.01, 0.02)	0.02	56.4%	15 (12, 17)	7 (5, 10)	7.5
Maldives	2016	64.4%	0.04 (0.01, 0.07)	−0.09 (−0.12, −0.06)	0.13	317.5%	11 (4, 18)	5 (−6, 16)	5.6
Mali	2018	46.6%	0.11 (0.09, 0.13)	0.05 (0.04, 0.06)	0.06	57.7%	29 (25, 33)	20 (15, 24)	9.5
Mozambique	2011	55.6%	0.15 (0.14, 0.17)	0.1 (0.09, 0.11)	0.05	32.5%	38 (35, 41)	22 (19, 25)	16.3
Myanmar	2015	51.0%	0.15 (0.14, 0.17)	0.05 (0.04, 0.06)	0.11	68.2%	40 (35, 44)	26 (21, 30)	13.8
Namibia	2013	64.6%	0.07 (0.04, 0.09)	0 (−0.02, 0.01)	0.07	106.2%	19 (12, 26)	−10 (−18, −2)	28.9
Nepal	2016	67.7%	0.07 (0.05, 0.09)	−0.02 (−0.04, −0.01)	0.09	134.3%	25 (20, 30)	3 (−3, 9)	21.9
Niger	2012	48.0%	0.15 (0.13, 0.17)	0.06 (0.05, 0.07)	0.09	62.3%	37 (34, 41)	35 (31, 39)	2.4
Nigeria	2018	33.9%	0.33 (0.32, 0.34)	0.11 (0.11, 0.11)	0.22	67.1%	54 (52, 56)	39 (37, 42)	14.9
Pakistan	2016	65.1%	0.15 (0.15, 0.16)	0.03 (0.02, 0.04)	0.12	80.9%	50 (47, 53)	35 (32, 39)	15.0
Papua New Guinea	2016	27.1%	0.26 (0.23, 0.28)	0.05 (0.04, 0.06)	0.21	81.6%	35 (31, 38)	28 (24, 31)	6.8
Peru	2012	63.2%	0.08 (0.07, 0.09)	0.02 (0.01, 0.03)	0.06	76.0%	27 (24, 30)	7 (3, 10)	20.5
Philippines	2017	58.9%	0.11 (0.09, 0.12)	−0.02 (−0.03, −0.01)	0.12	115.2%	37 (33, 41)	9 (4, 13)	28.2
Republic of Congo	2011	39.5%	0.20 (0.18, 0.22)	0.10 (0.09, −0.11)	0.10	50.0%	43 (39, 47)	16 (10, 22)	27.0
Rwanda	2019	92.1%	0.01 (0, 0.02)	0 (−0.01, 0.01)	0.01	80.0%	3 (1, 6)	2 (0, 5)	1.1
Senegal	2019	75.2%	0.06 (0.04, 0.08)	−0.06 (−0.08, −0.04)	0.12	200.0%	24 (19, 28)	17 (12, 22)	7.2
Sierra Leone	2019	63.3%	0.07 (0.05, 0.08)	0.01 (0, 0.01)	0.06	90.8%	20 (16, 24)	−1 (−5, 4)	20.3
South Africa	2016	48.0%	0.09 (0.06, 0.13)	0 (−0.01, 0.01)	0.10	103.2%	18 (11, 25)	−4 (−12, 4)	21.9
Tajikistan	2017	70.5%	0.07 (0.07, 0.07)	−0.05 (−0.05, −0.05)	0.12	173.2%	26 (22, 31)	−4 (−9, 1)	30.2
The Gambia	2020	48.0%	0.09 (0.08, 0.09)	0.02 (0.02, 0.02)	0.06	75.3%	22 (21, 23)	5 (4, 6)	16.8
Timor-Leste	2016	46.6%	0.16 (0.14, 0.17)	0.02 (0.01, 0.03)	0.14	86.0%	36 (32, 41)	19 (14, 24)	17.1
Togo	2013	61.7%	0.09 (0.07, 0.12)	0 (−0.01, 0.01)	0.09	103.3%	26 (21, 31)	5 (0, 10)	21.4
Uganda	2016	50.9%	0.09 (0.08, 0.11)	−0.04 (−0.06, −0.03)	0.14	147.8%	21 (18, 24)	−3 (−6, 1)	23.3
Yemen	2013	37.6%	0.24 (0.23, 0.25)	0.04 (0.03, 0.05)	0.20	83.3%	43 (41, 45)	39 (37, 41)	4.2
Zambia	2018	65.4%	0.06 (0.05, 0.07)	0.02 (0.02, 0.03)	0.03	58.6%	20 (16, 23)	8 (4, 12)	11.8
Zimbabwe	2015	68.0%	0.07 (0.05, 0.09)	0.02 (0.01, 0.03)	0.05	73.1%	22 (17, 27)	13 (8, 18)	9.0
Overall ^a^			0.13 (0.12, 0.15)	0.01 (0.00, 0.02)	0.12	92.0%	0.30 (0.27, 0.34)	0.14 (0.10, 0.18)	16.4

Note: ^a.^ 95% confidence intervals are presented in parentheses for estimated values. The overall averages in the last column are crude averages and not weighted by population size.

## Data Availability

The data used in this study are publicly available through the Demographic and Health Survey website.

## Appendix A. List of Countries and the Year of the Most Recent DHS

**Country**	**Year**
Afghanistan	2015
Angola	2015
Armenia	2015
Bangladesh	2016
Benin	2017
Burkina Faso	2010
Burundi	2016
Cambodia	2014
Cameroon	2012
Chad	2014
Comoros	2012
Republic of Congo	2011
Congo (DRC)	2013
Cote d’Ivoire	2012
Dominican Republic	2013
Egypt	2013
Ethiopia	2016
Gabon	2012
The Gambia	2020
Ghana	2014
Guatemala	2014
Guinea	2018
Haiti	2016
Honduras	2011
India	2020
Indonesia	2017
Jordan	2017
Kenya	2014
Kyrgyz Republic	2012
Lesotho	2014
Liberia	2019
Madagascar	2021
Malawi	2015
Maldives	2016
Mali	2018
Mozambique	2011
Myanmar	2015
Namibia	2013
Nepal	2016
Niger	2012
Nigeria	2018
Pakistan	2016
Papua New Guinea	2016
Peru	2012
Philippines	2017
Rwanda	2019
Senegal	2019
Sierra Leone	2019
South Africa	2016
Tajikistan	2017
Timor-Leste	2016
Togo	2013
Uganda	2016
Yemen	2013
Zambia	2018
Zimbabwe	2015

## Appendix B. List of DHS Variables Used in Multivariate Ranking

**Variable Name**	**Code**
Region	v101
Urban/Rural Status	v025
Maternal Education	v106
Wealth Quintile	v190
Sex of Child	b4
Health Insurance Coverage	v481
